# Real-life achievement of lipid-lowering treatment targets in the DIAbetes and LifEstyle Cohort Twente: systemic assessment of pharmacological and nutritional factors

**DOI:** 10.1038/s41387-018-0028-y

**Published:** 2018-04-25

**Authors:** Christina M. Gant, S. Heleen Binnenmars, Manon Harmelink, Sabita S. Soedamah-Muthu, Stephan J. L. Bakker, Gerjan Navis, Gozewijn D. Laverman

**Affiliations:** 1 Department of Internal Medicine/Nephrology, ZGT Hospital, Almelo and Hengelo, Hengelo, The Netherlands; 2 Division of Nephrology, Department of Internal Medicine, University Medical Centre Groningen, University of Groningen, Groningen, The Netherlands; 3 Centre of Research on Psychology in Somatic Diseases (CORPS), Department of Medical and Clinical Psychology, Tilburg University, Tilburg, The Netherlands; 4 Institute for Food, Nutrition and Health, University of Reading, Reading, UK

## Abstract

**Background/Objectives:**

Lowering low-density lipoprotein cholesterol (LDLc) in type 2 diabetes mellitus is of paramount importance in preventing cardiovascular disease. However, treatment targets for LDLc are often not reached. We studied the prevalence of LDLc target achievement in a real-life population of type 2 diabetes mellitus patients in secondary care, and investigated whether in those not on target, there is room for intensifying pharmacological and lifestyle management according to current treatment guidelines.

**Subjects/Methods:**

We performed a cross-sectional analysis in the DIAbetes and LifEstyle Cohort Twente-1 (DIALECT-1; *n* = 450, age 63 ± 9 years, 58% men, diabetes duration 11 (7–18) years). At baseline, we determined plasma LDLc concentration, pharmacological treatment (i.e., statin use), and lifestyle (physical activity and dietary intake). Patients were divided according to LDLc < 1.8, LDLc 1.8–2.5, and LDLc > 2.5 mmol/l. Dietary intake was collected from a validated Food Frequency Questionnaire (177 items) and we determined guideline adherence for different food groups. Physical activity was assessed with the Short Questionnaire to ASsess Health enhancing behavior.

**Results:**

LDLc data were available in 428 type 2 diabetes mellitus patients. LDLc ≤ 2.5 mmol/l was achieved in 317 patients (76%). In total, 76% of patients used statins, in those with LDLc > 2.5 mmol/l, this was 44%. Adherence to lifestyle guidelines was not different between the LDLc groups and was as follows: body mass index 6%, physical activity 59%, vegetables 7%, fruit 28%, legumes 59%, nuts 14%, dairy 19%, fish 36%, tea 8%, fats 66%, red meat 12%, processed meat 2%, alcohol 71%, sweetened beverages 34%, and sodium 12%.

**Conclusions:**

In type 2 diabetes mellitus patients in secondary health care, the target LDLc is achieved by three quarters of patients. Increasing statin treatment could be a first step to improve LDLc. In addition, there are ample opportunities for lifestyle management through increasing adherence to lifestyle guidelines.

## Introduction

Type 2 diabetes mellitus is associated with a substantially increased risk for cardiovascular disease (CVD), of up to two times higher than the general population, especially if disease duration is > 10 years^1, 2^. Prevention of cardiovascular complications is therefore one of the main aims in the overall treatment for type 2 diabetes mellitus, with appropriate treatment of dyslipidemia as one of the major goals. Lowering of low-density lipoprotein cholesterol (LDLc) in type 2 diabetes mellitus consistently reduces cardiovascular events^3–6^ and every 1.0 mmol/l reduction in LDLc is associated with a corresponding 20–25% reduction in CVD mortality and non-fatal myocardial infarction^7^.

Based on the literature, diabetes guidelines have incorporated targets for LDLc as well as recommendations for pharmacological and lifestyle treatment to reach these targets^8, 9^. However, several studies show that in clinical practice, a large proportion (44–67%) of patients with type 2 diabetes mellitus does not achieve the recommended treatment targets^10–12^, but an integral understanding of where opportunities may lie to improve management are lacking^13^. Whereas some studies on this subject focus on pharmacological management and general lifestyle habits (i.e., body mass index (BMI), smoking, and alcohol), and others on in-depth nutritional habits^14–16^, an integral approach is warranted, because both pharmacological treatment and dietary composition contribute to clinical outcomes and are part of clinical management. Previously, we have shown that for blood pressure management there are numerous opportunities in lifestyle management^17^.

In this study, we aim to determine the prevalence of LDLc target achievement in a real-life population of type 2 diabetes mellitus patients in secondary care, and investigate whether in those not on target, there is room for intensifying pharmacological and lifestyle management according to current treatment guidelines.

## Materials and Methods

We performed a cross-sectional analysis in baseline data from the DIAbetes and LifEstyle Cohort Twente-1 (DIALECT-1). The study population and study procedures of DIALECT-1 have been described previously^17^. DIALECT is an observational cohort study in patients with type 2 diabetes mellitus, which was designed to study associations between lifestyle habits and clinical outcomes. The study has been approved by local institutional review boards (METC-Twente, NL57219.044.16; METC-Groningen, 1009.68020), is registered in the Netherlands Trial Register (NTR trial code 5855), and is performed according to the guidelines of good clinical practice and the declaration of Helsinki as revised in 2008. All participants signed an informed consent form before participation. The reporting of the study conforms to the STROBE statement^18^.

### Setting

Between September 2009 and January 2016, a total of 450 high-risk type 2 diabetes patients were included in DIALECT-1, the flowchart of inclusion was previously described^17^. DIALECT-1 was performed in the outpatient clinic internal medicine of the Ziekenhuisgroep Twente (ZGT) Hospital, Almelo and Hengelo, the Netherlands. The ZGT hospital is a secondary care center for diabetes treatment. In the Netherlands, referral criteria to secondary health care are as follows: inability to achieve adequate glycemic control with oral antidiabetic drugs or a standard insulin regimen, overt nephropathy (macroalbuminuria and/or estimated glomerular filtration rate below 60 ml/min), or multiple cardiovascular complications.

### Participants

All patients, aged 18 + years, visiting the internal medicine outpatient clinic for type 2 diabetes mellitus treatment were eligible for the study. Exclusion criteria were inability to understand the informed consent procedure, insufficient command of the Dutch language, or renal replacement therapy. Eligible patients were selected from the electronic patient file and contacted by phone.

### Variables

At the clinic, sociodemographic characteristics, medical history, lifestyle behaviors, and current medications were recorded and anthropometric dimensions were measured using standard procedures. In clinical practice, upon initiation of statin therapy, nutraceutical use is extensively discussed as an alternative option to reduce LDLc, and thereafter is recorded in the electronic patient file. As we found no to very few mentions ( < 1%) of nutraceutical use in the patients’ files, nutraceutical use was not included in this study. Medical history was additionally reviewed in the hospital electronic patient files on three different occasions, by three different physician researchers. Macrovascular disease was defined as the presence of either coronary heart disease (CHD), cerebrovascular disease, or peripheral artery disease. CHD was defined as the presence of one of the following in medical history: physician diagnosed unstable angina pectoris, myocardial infarction, percutaneous coronary intervention, or coronary artery bypass graft. Cerebrovascular disease was defined as a history of transient ischemic attack or cerebrovascular accident. Peripheral artery disease was defined as the presence of one of the following in medical history: proven artery disease by angiogram or magnetic resonance angiogram, percutaneous transluminal angioplasty, or peripheral artery bypass graft.

Blood pressure was measured in a supine position by an automated device (Dinamap®; GE Medical Systems, Milwaukee, WI) for 15 min with a 1 min interval. The mean systolic and diastolic pressure of the final three measurements was used for further analysis.

Physical activity was assessed using the Short QUestionnaire to ASses Health enhancing physical activity questionnaire, which was previously validated and is commonly used in the Netherlands for population research^19^. Diet was assessed using a semi-quantitative Food Frequency Questionnaire (FFQ) inquiring about intake of 177 items during the last month, taking seasonal variations into account^20^. The FFQ was developed and validated at the Wageningen University and has been updated several times^20, 21^. For each item, the frequency was recorded in times per day, week, or month. The number of servings was expressed in natural units (e.g., slice of bread or apple) or household measures (e.g., cup or spoon). Both questionnaires were self-administered and filled out at home. The filled in questionnaires were checked for completeness by a trained researcher, and inconsistent answers were verified with the patients. Dietary data were converted into daily nutrient intake using the Dutch Food Composition Table of 2013^22^. Patients with a very low ( < 500 kcal/day) or very high ( > 6000 kcal/day) were excluded from the analyses, which was based on habitual caloric intake in the Netherlands^23^.

Blood was drawn from venipuncture in a non-fasting state, for measurement of cholesterol and other variables relevant for diabetes. Total high-density lipoprotein (HDL) cholesterol and triglycerides were determined with the enzymatic colorimetric method using routine laboratory procedures with a Clinical Chemistry Analyzer and Immunochemistry Analyzer (COBAS 8000; Roche Diagnostics GmbH, Mannheim, Germany). LDL cholesterol was calculated using the Friedewald formula (only if triglycerides < 4.5 mmol/l). 24 h urine collections were performed as prescribed previously^17^.

### Targets and definitions

The treatment target for LDLc was set as ≤ 2.5 mmol/l, according to the Dutch guidelines for cardiovascular risk management in type 2 diabetes mellitus used by internists^9^. As the European guideline for CVD prevention defines the target LDLc at < 1.8 mmol/l^8^ for patients with a very high risk (97% of our population), and this target is used by Dutch cardiologists, we also studied how well this target was reached.

According to the general Dutch guidelines for cardiovascular risk management used by internists, statin therapy is indicated when LDLc is > 2.5 mmol/l and the 10-year risk of CVD is ≥ 20%, or the risk is 10-20% and there is an additional risk factor (i.e. family member with CVD, physical inactivity, BMI ≥ 30, or reduced renal function)^9^. The 10-year risk is determined using age, smoking status, systolic blood pressure, and the total cholesterol/HDLc ratio. In type 2 diabetes mellitus patients, 15 years should be added to the patients’ age before calculating the risk. The first treatment step is simvastatin 40 mg/day, followed by atorvastatin 20–80 mg/day or rosuvastatin 1–40 mg/day, according to maximal tolerated doses and LDLc response.

In our study, medium intensity statin treatment was defined as follows: simvastatin 20–40 mg/day, atorvastatin 10–20 mg/day, rosuvastatin 5 mg/day, and pravastatin 40–80 mg/day^24^. Lower and higher prescribed dosages of the abovementioned statins were defined as low-intensity and high-intensity statin treatment, respectively.

General lifestyle recommendations were BMI ≤ 25 kg/m^2^ and smoking cessation^9^. The recommendation for physical activity was at least 5 days per week 30 min of moderate–vigorous exercise (such as cycling, brisk walking, and gardening)^9^. Dietary recommendations were derived from the Dutch dietary guidelines 2015 published by the Health Council of the Netherlands^25^, which are also adopted by the Dutch Diabetes Federation and used in clinical practice by dieticians treating diabetes patients^26^. In short, the recommended intakes were as follows: vegetables ≥ 200 g/day; fruits ≥ 200 g/day; wholegrain products ≥ 90 g/day; legumes ≥ 1 portion/week; unsalted nuts ≥ 15 g/day; low-fat dairy 2–3 portions/day (including milk or yoghurt); fish ≥ 1 portion/week; flack or green tea ≥ 3 cups/day; use soft margarines, liquid cooking fats and vegetable oils instead of butter or hard margarines and cooking fats; replace unfiltered coffee by filtered coffee; red meat ≤ 45 g/day; no processed meat; no consumption of sweetened beverages and fruit juices; alcohol ≤ 1 unit/day; sodium ≤ 2.3 g/day. As data on whether consumed grains were wholegrain or refined and data on whether consumed coffee was filtered or unfiltered were not available from the FFQ used in our study, these components were not analyzed here. Recommended daily legume and fish intake was calculated by dividing one portion size (60 g) by 7 and rounding up to 10 g/day. As the FFQ did not distinguish between salted and unsalted nuts, and type of tea, total nut intake, and total tea intake, respectively, were used in our calculations. Data on dietary sodium intake was derived from the 24 h urinary sodium excretion^17^.

### Statistics

All statistical analyses were performed using Statistical Package for the Social Sciences (IBM, Chicago, IL, USA), version 22.0. Normality of data was assessed by visually inspecting the frequency histograms. Normally distributed data were presented as mean ± SD. Skewed variables were expressed as median (interquartile range). Dichotomous variables were presented in number and percentage. Cases with missing data were excluded from the respective analyses. To describe characteristics of patients who had achieved different LDLc values, the population was divided in three groups according to LDLc < 1.8 mmol/l, 1.8–2.5 mmol/l, and > 2.5 mmol/l. Differences between the groups were tested using one-way analysis of variance (normally distributed), Kruskal–Wallis (skewed), or *χ*^2^-test (categorical).

## Results

### Baseline characteristics

For the current study, plasma LDLc concentrations were available in 428 patients of the total 450 patients included in DIALECT-1. Baseline characteristics are shown in Table 1. Mean age of patients was 63 ± 9 years and 58% of patients were men. The median diabetes duration was 11 (7–18) years, mean HbA1c was 57 ± 12 mmol/mol (7.4 ± 3.2%). Most patients were overweight or obese, mean BMI was 32.0 ± 6.3 kg/m^2^, and 6% of patients had a BMI < 25 kg/m^2^. The majority of patients had one or more complications: 65% of patients had microvascular disease, with nephropathy being the most frequent (42% of all patients), and 35% had macrovascular disease.

**Table 1:** Patient characteristics categorized by LDLc target groups

	Total population	LDLc < 1.8	LDLc 1.8–2.5	LDLc > 2.5	
*Number of patients (% of total population)*	*428*	*184 (43)*	*150 (35)*	*94 (22)*	P*-value*
Age, years	63 ± 9	64 ± 9	62 ± 9	62 ± 10	0.10
Male, *n* (%)	248 (58)	117 (64)	79 (55)	52 (52)	0.11
Duration of diabetes, years	11 [7–18]	13 [8–20]	11 [6–18]	10 [5–14]	0.006
Serum HbA1c, mmol/mol	57 ± 12	58 ± 11	56 ± 12	58 ± 13	0.12
Serum HbA1c, %	7.4 ± 3.2	7.5 ± 3.2	7.3 ± 3.2	7.5 ± 3.3	0.12
Insulin use, *n* (%)	271 (63)	129 (70)	88 (62)	54 (54)	0.02
Systolic blood pressure, mmHg	136 ± 16	136 ± 17	137 ± 16	136 ± 17	0.70
Diastolic blood pressure, mmHg	74 ± 10	73 ± 10	75 ± 9	75 ± 10	0.17
Heart frequency, beats/min	74 ± 13	73 ± 13	75 ± 12	74 ± 12	0.52
Antihypertensive treatment, *n* (%)	347 (81)	159 (86)	113 (79)	75 (74)	0.32
Total number of drugs	7 ± 3	7 ± 3	7 ± 3	6 ± 3	0.02
Microvascular disease, *n* (%)	280 (65)	135 (74)	85 (60)	60 (61)	0.008
Diabetic nephropathy, *n* (%)	178 (42)	90 (49)	50 (35)	38 (38)	0.02
Retinopathy, *n* (%)	103 (24)	59 (32)	32 (23)	12 (12)	0.001
Neuropathy, *n* (%)	155 (36)	70 (38)	42 (29)	43 (43)	0.03
Macrovascular disease, *n* (%)	149 (35)	78 (42)	38 (27)	33 (33)	0.007
Coronary heart disease, *n* (%)	93 (22)	49 (27)	22 (15)	22 (22)	0.04
Cerebrovascular disease, *n* (%)	46 (22)	25 (14)	13 (9)	8 (8)	0.26
Peripheral artery disease, *n* (%)	42 (10)	26 (14)	4 (3)	12 (12)	0.003
Total cholesterol, mmol/l	4.0 ± 0.9	3.3 ± 0.5	4.1 ± 0.4	5.0 ± 0.7	< 0.001
LDL-cholesterol, mmol/l	2.0 ± 0.8	1.4 ± 0.3	2.1 ± 0.2	3.1 ± 0.5	< 0.001
HDL-cholesterol, mmol/l	1.1 ± 0.3	1.1 ± 0.3	1.2 ± 0.4	1.1 ± 0.3	0.04
Total cholesterol/HDL ratio, mmol/l	3.8 ± 1.4	3.2 ± 1.0	3.7 ± 1.1	4.9 ± 1.7	< 0.001
Triglycerides, mmol/l	1.8 ± 0.9	1.9 ± 1.0	1.8 ± 0.8	1.9 ± 0.8	0.04
C-reactive protein, mg/l	2 [1–5]	2 [1–5]	2 [1–5]	3 [1–6]	0.13
*Pharmacological lipid-lowering therapy*					
Statin, *n* (%)	324 (76)	169 (92)	114 (76)	41 (44)	< 0.001
Ezetimibe, *n* (%)	34 (8)	9 (5)	10 (7)	15 (16)	0.004
Statin + ezetimibe, *n* (%)	22 (5)	8 (4)	6 (4)	8 (9)	0.16
Fibrate, *n* (%)	10 (2)	0 (0)	4 (3)	6 (6)	0.003
Statin + fibrate, *n* (%)	4 (1)	0 (0)	2 (1)	2 (2)	0.17
Other lipid-lowering therapy, *n* (%)	1 (0)	0 (0)	1 (1)	0 (0)	0.40
No lipid-lowering therapy, *n* (%)	87 (20)	14 (8)	31 (21)	42 (45)	< 0.001
Glucagon-like peptide-1 analogs, *n* (%)	43 (10)	14 (8)	20 (13)	9 (10)	0.22
*Lifestyle and nutritional factors*					
BMI, kg/m^2^	32.0 ± 6.3	33.0 ± 6.3	33.1 ± 6.0	32.4 ± 6.8	0.16
BMI ≤ 25 kg/m^2^, *n* (%)	24 (6)	7 (4)	8 (5)	9 (10)	0.14
Waist circumference, cm	112 ± 14	113 ± 14	112 ± 14	108 ± 14	0.02
Current smoker, *n* (%)	72 (17)	20 (20)	28 (15)	24 (17)	0.57
Adherence guideline physical activity, *n* (%)	244 (59)	97 (56)	92 (63)	55 (60)	0.46
Total caloric intake, kcal/day	1922 ± 636	1922 ± 645	1958 ± 628	1865 ± 632	0.56
Vegetables, g/day	98 [57–136]	95 [49–124]	99 [56–146]	101 [72–136]	0.26
Fruits, g/day	123 [66–227]	120 [66–218]	130 [73–226]	116 [66–232]	0.68
Legumes, g/day	12 [0–27]	17 [4–28]	11 [0–24]	11 [0–30]	0.34
Nuts, g/day	3 [0–9]	3 [0–6]	4 [0–11]	4 [0–10]	0.05
Dairy, g/day	213 [104–357]	231 [118–343]	207 [87–394]	167 [89–289]	0.10
Fish, g/day	10 [3–20]	10 [3–21]	11 [4–19]	10 [2–17]	0.72
Tea, g/day	71 [0–250]	71 [0–250]	71 [0–250]	125 [9–250]	0.12
Butter, hard margarines and cooking fats, g/day	0 [0–7]	0 [0–7]	0 [0–7]	0 [0–5]	0.73
Red meat, g/day	91 [66–117]	92 [69–117]	93 [69–125]	85 [58–110]	0.13
Processed meat, g/day	48 [30–72]	49 [30–73]	50 [33–70]	44 [26–65]	0.38
Sweetened beverages and fruit juices, g/day	27 [0–129]	21 [0–127]	27 [0–129]	29 [0–105]	0.88
Alcohol, units/month	5 [0–31]	3 [0–37]	8 [0–30]	4 [0–25]	0.50
Sodium, g/day	4.3 ± 1.8	4.2 ± 1.7	4.4 ± 2.0	4.1 ± 1.8	0.41

*BMI* body mass index, *LDLc* low-density lipoprotein cholesterol. Differences between the groups are determined using one-way ANOVA (normal distribution), Kruskal–Wallis (skewed distribution), or *χ*^2^-test (categorical variables)

Mean LDLc in the whole population was 2.0 ± 0.8 mmol/l. In total, 334 patients (78%) achieved the target LDLc ≤ 2.5 mmol/l, among which 184 patients (43% of the total population) achieved an LDLc < 1.8 mmol/l (Table 1). Patients with LDLc ≤ 2.5 mmol/l had a longer diabetes duration (*P* = 0.006) and more often used insulin (*P* = 0.02). Furthermore, patients who were on target LDLc ≤ 2.5 mmol/l more often had retinopathy (*P* = 0.001).

### Pharmacological lipid-lowering therapy and LDLc target achievement

Of all patients, 76% were on current statin therapy (Table 1). The most prevalent reasons for non-treatment were “not indicated according to guideline” and “previously reported side-effects/patient preference” (both 8% of the total population). In 7% of patients the reason for not using a statin was not documented in the patient file and there was no documentation of previous statin use. Of the 88 patients not on statin therapy, and with an indication for lipid-lowering therapy (LLT), 15 patients used ezetimibe and 6 patients used fibrates.

**Fig. 1:**
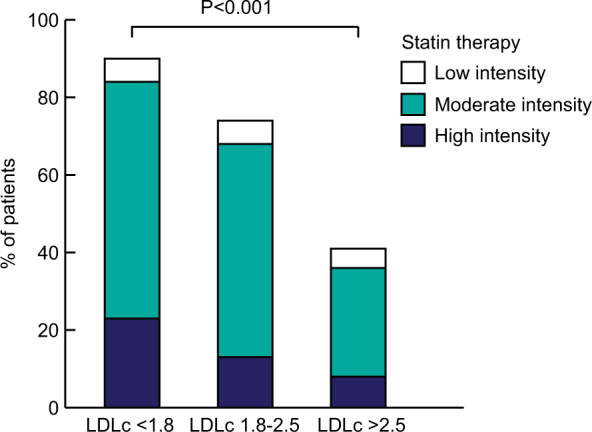
Intensity of statin treatment in LDLc groups. There was a significant difference in intensity of statin use between the LDLc groups. Medium-intensity statin treatment was defined as follows: simvastatin 20-40 mg/day, atorvastatin 10-20 mg/day, rosuvastatin 5 mg/day, and pravastatin 40-80 mg/day. Lower and higher prescribed statin dosages were defined as low intensity and high intensity, respectively

Of patients on target LDLc ( ≤ 2.5 mmol/l), 83% used statins, whereas 41% of patients with an LDLc > 2.5 mmol//l used statins. High-intensity statin treatment was the most prevalent in patients with a LDLc < 1.8 mmol/l (23%) versus 13% in those with LDLc 1.8–2.5 mmol/l and 8% in those with LDLc > 2.5 mmol/l (Fig. 1). Of patients who did not achieve the target LDLc of ≤ 2.5 mmol/l, 46% did not use any LLT, whereas 5% used low-intensity and 28% used moderate-intensity statin treatment. Of the 54 non-statin users in the LDLc > 2.5 mmol/l group, 24 patients experienced side effects or had a personal preference to avoid statins, and in 23 patients the reason for not using statins was not documented.

### Lifestyle and LDLc target achievement

Adherence to general lifestyle guidelines was low in the overall population and was not different between the LDLc target groups (Table 1). In the overall population, 6% had a BMI ≤ 25 kg/m^2^ and 59% had physical activity as recommended. Smoking guidelines were followed relatively well, as 83% were either non-smokers or former smokers.

The mean total kilocaloric (kcal) intake per day was 1922 ± 636 kcal/day and was not different between the LDLc target groups (Table 1). There were no differences in absolute intake of dietary products among the LDLc groups. Median vegetable intake was 98 [57–136] g/day and median fruit intake was 123 [66–227] g/day.

**Fig. 2:**
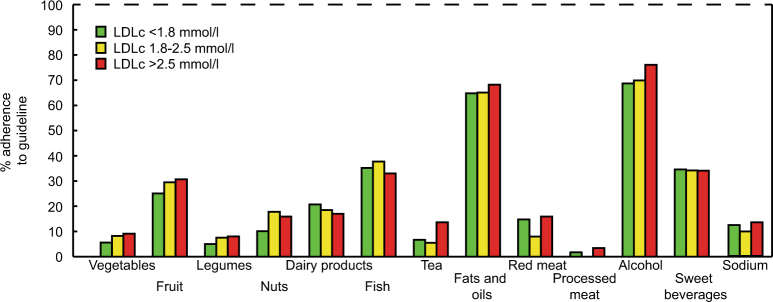
Adherence to dietary guidelines on nutritional intake. There were no differences in adherence rates between the LDLc target groups

Adherence to dietary guidelines was low in the whole population and there were no differences between the LDLc target groups (Fig. 2). Only 7% of the population consumed ≥ 200 g vegetables per day, whereas 28% consumed ≥ 200 g fruit per day. Furthermore, 59% of the population consumed legumes once weekly, 14% ate ≥ 15 g nuts per day, and 19% consumed 2–3 portions of low-fat dairy per day. Fish intake was as recommended in 36% of the population and 8% drank tea as recommended. Adherence to fats and oils intake was reasonably well with 66%. In regard to meat consumption, 12% did not eat more red meat than recommended and 2% ate no processed meats. Alcohol intake was one unit per day or less in 71%, and 34% drank no sweetened beverages. Sodium intake was below 2.3 g/day in 12% of the population.

## Discussion

In this real-life study in type 2 diabetes mellitus with high cardiovascular risk, the target LDLc of ≤ 2.5 mmol/l is reached in three quarters of the patients. Statin use was markedly more frequent and statin dosage was higher in patients who had achieved the LDLc target. Therefore, pharmacological treatment with statins, used in approximately three quarters of patients, is the most important part of LDLc lowering treatment in routine clinical practice. In contrast, adherence to lifestyle guidelines was poor in the whole study population, especially on BMI and intake of vegetables, legumes, nuts, red and processed meat, tea, and sodium.

Overall, the data illustrate that statin therapy is well incorporated in routine diabetes care and is an effective tool to reach target LDLc in the real-world setting of type 2 diabetes mellitus treatment. The percentage of adequately controlled LDLc reported here is somewhat higher than found in other studies. A previous study demonstrated LDLc target achievement in 56% of type 2 diabetes mellitus patients treated in the primary health care setting in the Netherlands^11^. In the large European EUROASPIRE study, LDLc targets attainment was reported in 33% of patients^12^. De Cosmo at al.^10^ reported adequately controlled LDLc in 51% of patients with diabetes and chronic kidney disease. The high percentage of patients on target for LDLc we found illustrates that LLT is well incorporated in routine secondary clinical care of high risk type 2 diabetes mellitus patients. The target LDLc was more often reached by patients with more serious disease, as indicated by a longer duration of type 2 diabetes mellitus, more frequent insulin use, and higher prevalence of microvascular complications. This suggests that a higher urgency for aggressive LDLc lowering treatment is experienced in patients with a higher grade of comorbidity.

In the patients who were not on statin treatment, one-third had no strict indication for LLT, one-third had previously experienced side effects, and in roughly one-third, the reason for not using a statin was not documented. Possibly, the latter subgroup consists of individuals with a preference of not using a statin, either based on general perceptions or because of side-effects in the past (this was not documented in the patient files). The chance that the option of prescribing a statin has been overlooked is negligible, taking into account the disease duration, frequent contact with sequential physicians and diabetes nurses, and a system in which also pharmacists verify adherence to guidelines.

Adherence to dietary recommendations was low in our population of type 2 diabetes mellitus patients. It should be noted that the majority of findings on dietary intake reported here were not different from dietary intake in the general Dutch population as reported by the National Institute for Public Health^27–29^. The general population had a slightly lower intake of fruits (113 vs. 123 g/day in DIALECT), legumes (5 vs. 12 g/day in DIALECT), red meat (79 vs. 91 g/day in DIALECT), and low-fat dairy (180 vs. 213 g/day in DIALECT). Intake of processed meat (50 vs. 49 g/day in DIALECT) and fish (18 vs. 10 g/day in DIALECT) was comparable. Intake of vegetable was higher in the general population, 139 g/day vs. 98 g/day in DIALECT. Interestingly, intake of sweetened beverages was substantially higher in the general population (336 vs. 27 g/day in DIALECT). The difference in intake of sweetened beverages possibly reflect the effect of dietary counselling in diabetes patients. In conclusion, it is important to recognize that non-adherence to dietary guidelines is not a problem specific for type 2 diabetes mellitus patients, but is a population-wide phenomenon.

It should be noted that the LDLc target in Dutch secondary care is defined as ≤ 2.5 mmol/l, both for high-risk and very high-risk patients^9^. This is different from the European guidelines, where the LDLc target is < 1.8 mmol/l for very high-risk patients (97% of our population)^8^. When using European guidelines, target achievement was somewhat lower (43%). However, when using these guidelines, results of analyses on where treatment opportunities lie to improve target achievement were similar: in patients not on target, high intensity statin use was infrequent ( < 15%) and adherence to lifestyle guideline adherence was low in all LDLc groups.

On a side note, we found a relatively high grade of adherence to the recommendation for physical activity (59%), in the general Dutch population this number was 54% in 2015^30^. In a systematic review, correlations between subjectively and objectively measured physical activity were weak to moderate^31^. This poses the question whether the compliance to the recommendation we report here is an accurate reflection of the actual physical activity of our population.

This study has several strengths. The integration of pharmacological and lifestyle parameters collected in a real-life setting provides the best possible tool to define opportunities to improve treatment strategies in type 2 diabetes mellitus. In addition, our study population represents a real-life clinical setting, where inclusion bias was minimal due to the broad inclusion criteria. A potential limitation of the study is that venipuncture was not performed in the fasting state^8^, leading to a possible overestimation of serum triglycerides and therefore underestimation of LDLc levels. However, serum triglycerides were not higher than expected and therefore we estimate that this effect was minimal. In addition, the use of the FFQs might lead to underestimation of intake of unhealthy products in dietary intake. Nevertheless, there are currently no better methods for registration of dietary habits in a study with this size. Lastly, the cross-sectional design only allows to study associations, and not causality. Future prospective studies are necessary to evaluate the effects of increasing pharmacological treatment and increasing lifestyle guideline adherence on LDLc target achievement.

What should be the implications of this study? We found that pharmacological treatment with statins was substantially higher in patients who had reached the LDLc target. Therefore, in patients who are not on target LDLc, the first step could be to explore the opportunities to either start or intensify statin treatment. As opposed to blood pressure management in this cohort, where therapy resistance is an issue in roughly 40% of the patients not on target blood pressure^17^, our data suggest that resistance to statin treatment is less common. In those with an LDLc not on target, only 8% were on high-intensity statin treatment. High-intensity statin treatment can reduce LDLc by 40–60%, vs. a 20–30% reduction on low-intensity treatment^32^. Therefore, intensifying statin treatment, either by increasing the dose, or by switching to a more potent compound, could be an option in a large subset of patients not on target. Furthermore, adherence to therapy should be addressed. Relatively low adherence to statin therapy has been previously reported^33–35^, especially after negative reports on statins by the mainstream media^36, 37^, rendering it worthwhile to investigate whether prescribed statins are actually ingested by the patients. Unfortunately, such data were not available here. It should be noted that statin treatment efficacy can vary considerably per individual, in literature a range of 5–70% LDLc reduction in different individuals is reported^38, 39^. Statin treatment resistance, i.e., not achieving LDLc target, despite high-intensity statin treatment, has been associated with black racial ancestry, polymorphisms in genes affecting statin pharmacodynamics and pharmacokinetics, smoking, inflammation, and HIV infection^40^. Certain drugs may also reduce statin effectiveness by reducing bioavailability (i.e., bile acid sequestrants) or increasing statin metabolism (i.e., rifampicin). Nutraceuticals, which sometimes are used in the general population, might also influence LDLc target achievement; however, few studies have been performed on the interaction between statin use and nutraceuticals, and therefore the effect of nutraceuticals statin efficacy remains unclear^41^. In addition, clinical conditions that increase cholesterol levels, such as hypothyroidism, should also be reviewed. However, in a real-life setting, LDLc target non-achievement, despite statin treatment, can most often be attributed to treatment non-adherence rather than treatment resistance^42, 43^.

For patients who fail to reach their LDLc target, there are alternative pharmacological options to reduce LDLc. First, in patients who do not achieve the target LDLc despite maximum-tolerated dose statin use, ezetimibe therapy should be considered^8, 44^. We found that only a quarter of not on target patients used ezetimibe, with or without concurrent statin use, and therefore ezetimibe therapy could be increased. In the case of mixed dyslipidemia (i.e., high LDLc and high triglycerides), fibrate therapy, which was used by 8% of patients not on LDLc target in this population, could also be considered^45^. When both maximum-tolerated statin use and ezetimibe are insufficient, the relatively new drug class of PCSK9 inhibitors (not used in this population) has the potential to reduce LDLc by 32–71%, depending on the used dosage^46, 47^. In addition, glucagon-like peptide-1 (GLP-1) analogs, which improve glycemic regulation, body weight, and blood pressure in type 2 diabetes mellitus, have also been shown to reduce LDLc and triglycerides, and increase HDLc^48–50^. Moreover, GLP-1 analogs have a favorable effect on total cardiovascular risk: in the recent LEADER trial, cardiovascular death was 22% lower in type 2 diabetes mellitus patients treated with liraglutide, compared to placebo-treated patients^51^. Therefore, the use of GLP-1 analogs in clinical practice should not be overlooked, especially in those with poor glycemic regulation in combination with obesity, hypertension, or dyslipidemia^52^.

Alternatively, especially in the patients that have a personal preference not to use statins or are intolerant to statins, lifestyle intervention could be a worthwhile option to improve LDLc and improve general cardiovascular risk management. With respect to diet, one could consider to focus on increasing the intake of vegetables and legumes, and reducing the intake of red and processed meat. It has previously been shown in patients not on statin therapy, dietary changes can reduce LDLc by 13–30%^53–55^. In a post-hoc analyses of the Alpha Omega Trial, in which the effect of omega 3 fatty acid supplementation on cardiovascular outcomes was studied in post-myocardial infarction patients, a beneficial effect was shown in those not on current statin treatment^56^. It should be noted that little data is available on the extent of LDLc reduction through dietary changes in patients already on statin treatment. Stakeholders of nutritional research in the Netherlands have hypothesized that the high efficacy of pharmacological therapy is one of the reasons that lifestyle intervention is less emphasized in clinical care^57^. This is supported by our finding that statins, and not lifestyle, are the main determinants of LDLc control. Nevertheless, adopting a healthy lifestyle has pleiotropic effects not only on cholesterol, but also on other cardiovascular risk factors such as obesity, blood pressure, and insulin resistance, for which treatment resistance is a growing concern^58–63^. For example, a recent meta-analysis of 39 studies has shown that physical exercise can reduce LDLc and improve insulin sensitivity^64^, especially in obese subjects. However, adherence to dietary guidelines on these compounds is low in the overall population, illustrating that community intervention might be more appropriate, then just targeting type 2 diabetes mellitus patients. In the meantime, focus should be placed to promote a healthy diet and lifestyle in patients with type 2 diabetes mellitus.

## Conclusion

In this population of high-risk type 2 diabetes mellitus patients in a real-world secondary health care, the LDLc target is achieved by the majority of the population. High-intensity statin treatment was infrequent in patient who did not achieve the LDLc target; therefore, a good opportunity to improve LDLc target achievement could be to put as many as possible patients on appropriate dose of statins. Nevertheless, adherence to lifestyle and dietary recommendation is low, especially on BMI and intake of vegetables, legumes, nuts, red and processed meat, tea, and sodium. Therefore, the focus on lifestyle intervention remains of great importance, because of multiple beneficial effects on obesity, blood pressure, and insulin resistance.
